# Similar thrombolysis outcomes in acute stroke patients with and without atrial fibrillation if pre-stroke CHA_2_DS_2_-VASc score is low: A retrospective study

**DOI:** 10.1097/MD.0000000000018680

**Published:** 2020-01-10

**Authors:** Hung-Ming Wu, Chih-Ping Chung, Yung-Yang Lin

**Affiliations:** a Institute of Brain Science; b Brain Research Center; c Institute of Clinical Medicine; d Department of Critical Care Medicine; e Department of Neurology, Neurological Institute, Taipei Veterans General Hospital; f School of Medicine, National Yang-Ming University, Taipei, Taiwan; g Department of Neurology, Taipei Hospital, Ministry of Health and Welfare, New Taipei City, Taiwan.

**Keywords:** acute ischemic stroke, atrial fibrillation, CHA_2_DS_2_-VASc score, outcome, recombinant tissue plasminogen activator

## Abstract

The prognosis of acute ischemic stroke patients treated with intravenous (IV) recombinant tissue plasminogen activator (rtPA) is poorer in patients with atrial fibrillation (AF) than patients without AF, which might be related to the greater stroke severity in AF patients. Higher pre-stroke CHA_2_DS_2_-VASc scores are associated with greater stroke severity and poorer outcomes. AF Patients tend to have higher CHA_2_DS_2_-VASc scores than the non-AF patients. We thus hypothesized that pre-stroke CHA_2_DS_2_-VASc scores can be used to improve outcome stratification of IV thrombolysis therapy in acute stroke patients with and without AF. We retrospectively enrolled ischemic stroke patients who received IV-rtPA and categorized them into 2 groups: low-risk (CHA_2_DS_2_-VASc scores ≤ 2) and high-risk (CHA_2_DS_2_-VASc scores ≥ 3) groups. We compared the outcomes between AF and non-AF patients and the interactive effects of the levels of CHA_2_DS_2_-VASc scores on this outcome difference. In the low-risk group, there was no difference in outcomes between the AF and non-AF patients. In the high-risk group, the AF patients had worse outcomes at 3 and 6 months. Our results suggest that pre-stroke CHA_2_DS_2_-VASc scores are a useful outcome predictor of IV thrombolytic therapy in acute stroke patients with AF.

## 1 Introduction

Intravenous (IV) recombinant tissue plasminogen activator (rtPA) is an effective treatment for patients with acute ischemic stroke, but it also increases the risk of symptomatic intracranial hemorrhage (sICH).^[1]^ In 1 meta-analysis study, patients with atrial fibrillation (AF) have higher risk of sICH and less favorable outcome after IV thrombolysis compared with non-AF patients.^[2]^ In another study, AF, stroke severity, and age were all correlated with sICH after IV thrombolysis.^[3]^ The greater stroke severity indicated by higher National Institutes of Health Stroke Scale (NIHSS) scores is probably accountable for this poor outcome after IV thrombolysis in AF patients.^[4,5]^ However, the reported effects of baseline NIHSS scores on the outcomes for such patients are not consistent, as another study reported that patients with AF and higher baseline NIHSS scores had good functional outcomes following IV-rtPA.^[6]^ Therefore, there may be other tools that could provide more accurate outcome stratification for acute stroke patients with AF after IV-rtPA.

AF increases the risk of stroke by 5 times,^[7]^ and stroke risk also increases with increases in CHA_2_DS_2_-VASc (Congestive heart failure, Hypertension, Age ≥75 years, Diabetes, previous Stroke or transient ischemic attack, Vascular disease, Age 65–74 years, Sex category) scores.^[8]^ AF-associated strokes are more severe and more often lead to disability and death.^[9,10]^ For patients with AF, higher pre-stroke CHA_2_DS_2_-VASc scores also predict more severe stroke symptoms^[11]^ and poor post-thrombolysis prognosis.^[12]^ For both AF and non-AF patients, higher pre-stroke CHA_2_DS_2_-VASc scores are associated with reduced chances of good functional outcomes and increased incidences of mortality at 90 days.^[11,13]^ In cases of acute ischemic stroke, the finding that AF patients have poorer post-stroke outcomes than those without AF is probably due to those AF patients having a greater proportion of high CHA_2_DS_2_-VASc scores.^[13]^ However, for non-AF patients, the post-thrombolysis outcomes according to CHA_2_DS_2_-VASc scores and the difference in outcomes compared to patients with AF are unclear.

Therefore, we hypothesized that

1. the poor outcomes in AF patients after IV-rtPA are associated with a greater CHA_2_DS_2_-VASc scores, and
2. different outcomes for patients with AF and patients without AF are only present among those with high-risk CHA_2_DS_2_-VASc scores while not being present in those with low-risk CHA_2_DS_2_-VASc scores.

The aim of this study was to investigate the relationship between pre-stroke CHA_2_DS_2_-VASc scores and the difference in outcomes following IV thrombolysis among patients with AF and patients without AF.

## 2 Materials and methods

### 2.1 Study population

We retrospectively enrolled acute ischemic stroke patients with AF and acute ischemic stroke patients without AF who received IV thrombolytic therapy at the Neurological Institute of Taipei Veterans General Hospital between January 2012 and November 2017. The inclusion criteria were as follows:

1. age ≥ 18 years;
2. diagnosis of acute ischemic stroke;
3. no contraindication for thrombolysis therapy; and
4. IV thrombolysis within 4.5 hours of onset of symptoms. The exclusion criteria were:
  1. pre-stroke modified Rankin scale (mRS) ≥ 1, and
  2. patients who also received intra-arterial thrombolytic therapy.

The diagnosis of AF was based on a 12-lead electrocardiogram and 24-hour Holter monitoring or a history of paroxysmal AF. According to the CHA_2_DS_2_-VASc scores, which are based on the presence/absence of each risk factor of stroke, we divided the patients into a low-risk group (CHA_2_DS_2_-VASc scores ≤ 2) and a high-risk group (CHA_2_DS_2_-VASc scores ≥ 3). This study was approved by the local ethics committee of Taipei Veterans General Hospital.

### 2.2 Clinical assessment

The clinical data included age, gender, history of hypertension, diabetes mellitus, congestive heart failure/left ventricular dysfunction, previous stroke or transient ischemic attack, vascular disease (prior myocardial infarction, peripheral artery disease, or aortic plaque), use of anti-platelet or anti-coagulation therapy prior the time of stroke onset, NIHSS score prior to thrombolytic therapy and discharge, the time interval between stroke onset and thrombolytic therapy, and the rtPA dose (the ratio of dose/body weight). Computed tomography (CT) was performed routinely before IV thrombolytic therapy (within 4.5 hours of the onset of symptoms) and was performed 24 hours after IV thrombolytic therapy, and the Alberta Stroke Program Early CT Score (ASPECTS) was used to assess the severity of the ischemic region in the brain CT.^[14]^

### 2.3 Outcome measurements

The outcome analysis was evaluated by the presence/absence of intracranial hemorrhage (ICH) or sICH during admission, all-cause mortality, and the Barthel index/functional outcome at discharge, 1 month, 3 months, and 6 months after stroke onset. A sICH was defined as any type of ICH with increased an NIHSS score of more than 4 or leading to death or surgery within 7 days.^[15]^ The functional outcome was assessed by mRS, with a favorable outcome defined as a mRS score ≤ 1.

### 2.4 Statistical analysis

We used the Windows SPSS package (version 20.0, IBM Corp.) to perform the statistical analysis. Categorical variables were compared using the chi-square test, and the Kolmogorov–Smirnov test was used to evaluate the data distribution. The continuous variables were analyzed by independent *t* test or Mann–Whitney *U* test to examine the difference between patients with AF and patients without AF. All numerical data are presented as the mean ± standard deviation (SD). We used the multivariate logistic regression to estimate the independent predictors of favorable outcome and ICH for patients with AF compared to patients without AF by calculating the odds ratio (OR) with two-sided 95% confidence intervals (CI) for outcome measures. A *P* value < .05 was considered statistically significant.

## 3 Results

### 3.1 Baseline characteristics

A total of 198 acute ischemic stroke patients who received IV thrombolysis were included in the study, and 1 patient with pre-stroke mRS ≥ 1 and 25 patients who also received intra-arterial thrombolytic therapy were excluded. Finally, 172 cases were analyzed. 69 patients (14 patients with AF and 55 patients without AF) were enrolled in the low-risk group (CHA_2_DS_2_-VASc scores ≤ 2), and 113 patients (39 patients with AF and 64 patients without AF) were enrolled in the high-risk group (CHA_2_DS_2_-VASc scores ≥ 3). The proportion of patients with AF included in the high-risk group (CHA_2_DS_2_-VASc scores ≥ 3) was greater that the proportion of patients without AF included in the high-risk group (AF vs non-AF: 73.6% vs 53.8%; *P* = .018). The average age of the patients with AF was higher than that of the patients without AF, but there was no gender difference between the AF patients and non-AF patients in either the low-risk or high-risk group (Table 1). In the low-risk group, the mean systolic blood pressure and the mean dose of rtPA were lower in the patients with AF than the patients without AF, but the prevalence of using oral anticoagulants and the mean International normalized ratio (INR) of prothrombin time were higher in the patients with AF. In the high-risk group, the rtPA dose was lower in the patients with AF than the patients without AF, but the prevalence of using anticoagulants was higher in the AF patients. There were no differences in the baseline NIHSS scores between the patients with AF and the patients without AF in either the low-risk group or the high-risk group. The discharge NIHSS scores in the high-risk group were higher in the patients with AF than the patients without AF, but they were no different in the patients with AF and the patients without AF in the low-risk group (Table 1).

**Table 1:**
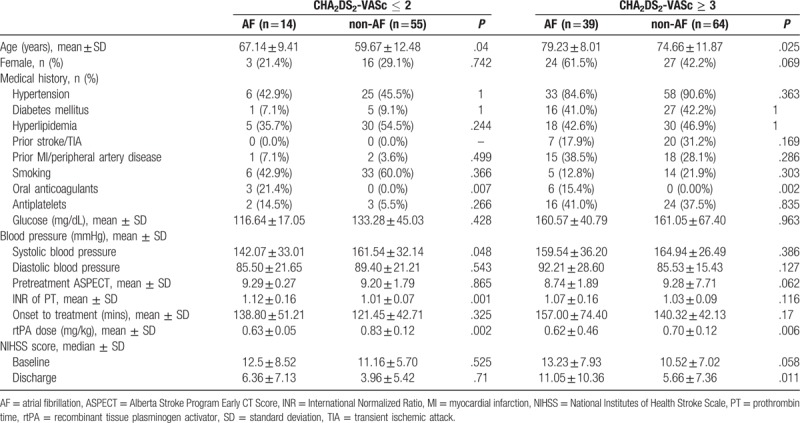
Clinical characteristics of patients with cerebral infarction receiving intravenous thrombolysis: classification by CHA_2_DS_2_-VASc score and atrial fibrillation.

### 3.2 Outcome and multivariate analysis

In the high-risk group, the risk of ICH in patients with AF was higher than that in patients without AF, but the risks of sICH and mortality were similar in both groups of patients. In the low-risk group, there was no difference between the 2 groups of patients in terms of the risks of ICH, sICH, and mortality. In addition, the proportion of favorable functional outcomes and the scores of the Barthel index in the low-risk group were similar between the patients with AF and the patients without AF. However, in the high-risk group, the patients with AF had fewer favorable functional outcomes at 3 and 6 months and had lower Barthel index scores at discharge, 1 month, 3 months, and 6 months, than the patients without AF (Table 2).

**Table 2:**
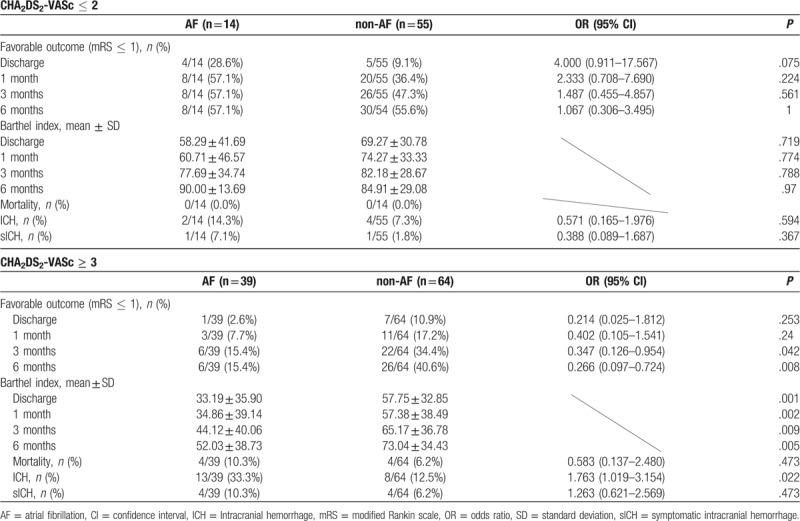
Clinical outcomes of patients with cerebral infarction receiving intravenous thrombolysis: classification by CHA_2_DS_2_-VASc scores and atrial fibrillation.

In the multivariate analysis with adjustments for age, rtPA dosage, and AF, the baseline NIHSS scores were significantly associated with favorable outcomes at 6 months in the patients in the high-risk group (Table 3). In addition, in the multivariate logistic regression analysis using AF, age, systolic blood pressure, and ASPECT scores as the variables, AF and ASPECT scores were shown to be independent predictors of ICH in the patients in the high-risk group (Table 4).

**Table 3:**
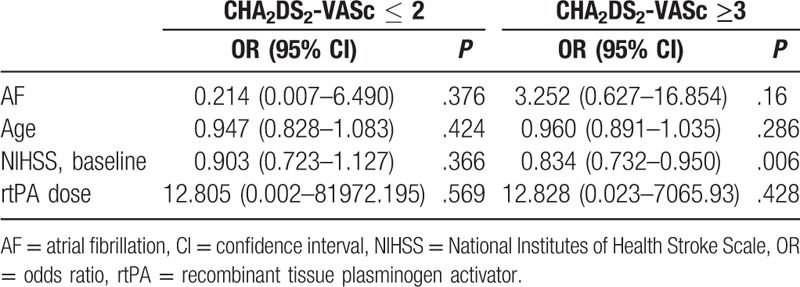
Multivariate logistic regression to predict favorable outcome (modified Rankin scale ≤ 1) at 6 months: classification by CHA_2_DS_2_-VASc score.

**Table 4:**
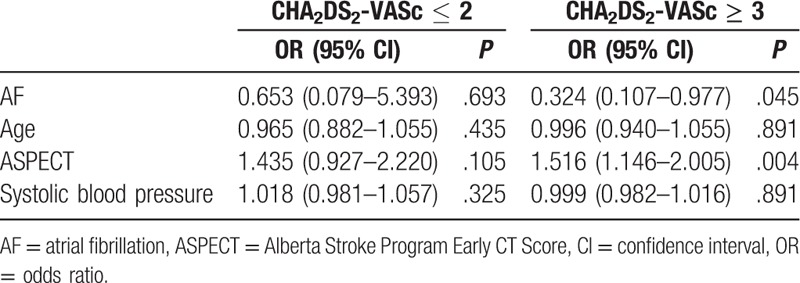
Multivariate logistic regression to predict intracranial hemorrhage: classification by CHA_2_DS_2_-VASc score.

## 4 Discussion

In the present study, we demonstrated 2 major findings regarding patients who received IV-rtPA therapy. First, in the high-risk group, there were fewer favorable outcomes (mRS ≤ 1) and higher ICH risk in the patients with AF compared to those without AF, but there was no difference in the rates of sICH and mortality. Second, in the low-risk group, there was no difference between the AF patients and non-AF patients in the rates of ICH, sICH, and mortality, or the proportion of favorable outcomes.

High NIHSS scores could be associated with poorer outcomes and a lack of recovery in the Barthel index after cerebral ischemia.^[16]^ Indeed, this study found that the AF patients with higher NIHSS scores had fewer favorable outcomes and a lower mean Barthel index score than those without AF in the high-risk group; however, in the low-risk group, the NIHSS scores were not associated with any difference between the AF patients and non-AF patients, including in terms of prognosis. The results for the high-risk group were thus consistent with the findings of previous studies,^[4,5,17]^ while the results for the low-risk group were not. This may have been because those previous studies did not classify patients according to the CHA_2_DS_2_-VASc score. The increased CHA_2_DS_2_-VASc scores in patients with AF are associated with increased NIHSS scores^[18]^ and little improvement in acute neurologic deficits.^[19]^ Patients with higher CHADS_2_ scores have higher levels of systemic inflammatory markers, such as serum C-reactive protein,^[20]^ which could be associated with their poorer neurological outcomes.^[21]^ In comparison with sinus rhythm, the vascular endothelial function is poorer in AF, and endothelial function is negatively correlated to the increase of CHA_2_DS_2_-VASc scores.^[22]^ The enhancement of endothelial function inhibits thrombus development.^[23]^ The prevalence of intracardiac thrombus/sludge was increased with increasing CHADS_2_ scores,^[24]^ and the presence of intracardiac thrombus/sludge might result, in turn, in larger infarction sizes and higher NIHSS scores.^[25]^ The incidence of proximal artery occlusion in acute stroke is also increased with higher CHA_2_DS_2_-VASc scores.^[26]^ These above observations showed that clinical management of stroke in AF patients is much more challenging in the high-risk than the low-risk group. Conversely, in the low-risk group, the similar NIHSS scores and outcomes between the 2 groups of patients may indicate that the AF and non-AF patients were exposed to comparable low-risk situations.

In this present study, in both low-risk and high-risk groups, the AF patients received a lower dose of thrombolytic agents, on average, than non-AF patients. In the low-risk group, the use of smaller doses in the patients with AF may have been useful for ensuring the safety and efficacy of the thrombolytic agents. The findings in the low-risk group were consistent with previous findings indicating that the chance of cerebral hemorrhage is decreased with dose reduction even as the improvement in symptoms is not decreased with the decrease in dosage.^[27,28]^ However, in the high-risk group, the use of smaller doses in the patients with AF may have reduced the effectiveness of the thrombolytic agents, which is suggested by the observation that the patients with AF had more severe symptoms at discharge than those without AF in spite of having similar symptoms upon admission. Elevated levels of several systemic inflammatory markers have previously been found to be significantly associated with an increased risk of thromboembolic events in patients with AF.^[29]^ Higher inflammatory conditions may also be associated with increased thrombin generation and related to early recurrence of ischemic lesion.^[30]^ In the high-risk group in this study, it is possible that the smaller doses of rtPA in the patients with AF may have had difficulty in overcoming the higher inflammatory and hypercoagulable conditions of the AF patients in order to achieve and maintain successful thrombolysis.

Another possible explanation for why the AF patients had worse outcomes in the high-risk group is that they were older than the non-AF patients. The incidence rate of AF is strongly associated with age, which is approximately 10 to 20/1000 person-years in the age group 65 to 74 years and increases to approximately 20 to 40/1000 person-years in the age group 75 to 84 years.^[31,32]^ Age has been shown to be an important clinical predictor of mortality and lack recovery of the Barthel index in patients who have suffered from acute ischemic stroke.^[16]^ In both the high-risk and low-risk groups in this study, the AF patients were older than the non-AF patients, which was consistent with the findings of the previous literature.^[5,17,33]^ However, the negative effects of age on prognosis were not significant among the low-risk group patients in this study. The reason for this might be that smaller doses of thrombolytic agents are beneficial for older patients.^[34]^

Although there were no differences in the rates of sICH and mortality between the patients with AF and the patients without AF in either the high-risk or the low-risk group in the present study, the patients with AF in the high-risk group, but not the low-risk group, had a higher ICH risk than the patients without AF. On the other hand, AF and ASPECT scores were correlated with ICH in the high-risk group. These findings for the high-risk group were consistent with those of previous studies indicating that cerebral hemorrhage is associated with AF^[3]^ and lower ASPECT scores.^[35]^ It could be that the components of the CHA_2_DS_2_-VASc score, such as older age and history of previous stroke and hypertension, are independent risk factors associated with increased risk for a bleeding event in patients with AF.^[36]^ Therefore, the AF patients in the low-risk group in this study had fewer of these risk factors and might have thus had a relatively reduced chance of cerebral hemorrhage.

Several limitations were found in the present study. First, this was a retrospective, single hospital study. Second, some selection bias could have occurred due to the fact that any patients who received IV-rtPA and then underwent further intra-arterial thrombolysis were excluded. Third, the population in this study was relatively small. Fourth, this study did not classify the non-AF patients into subgroups according to the subtype of stroke (i.e., large artery occlusion or small vessel occlusion). Fifth, the lower rtPA dose in our AF patients than non-AF patients might be a confounding factor. The explanation might be that the neurologists were not totally blinded to the past history of AF before deciding the dose of rtPA.

## 5 Conclusions

The present study indicates that the stratification of the CHA_2_DS_2_-VASc score for AF patients not only predicts the risk of stroke but also predicts the neurological outcomes following IV thrombolytic therapy. Compared to the patients without AF, the outcomes of IV-rtPA treatment in the patients with AF were stratified by the CHA_2_DS_2_-VASc scores. In the low-risk group, there was no difference in outcomes between AF and non-AF patients, which might be due to that the AF and non-AF patients were exposed to comparable low-risk situations. In contrast, in the high-risk group, the AF patients had poorer outcomes, which might be related to the higher systemic inflammatory status, poorer endothelial function, higher prevalence of intracardiac thrombus/sludge formation, and higher incidence of proximal artery occlusion. Therefore, the treatment strategies could be differentially used according to the levels of CHA_2_DS_2_-VASc score. In the high-risk group, dose adjustment, anti-inflammatory treatment, or intra-arterial thrombolysis are more likely considered in AF patients, although further investigations are needed.

## Acknowledgments

We are very grateful for the Taipei Veterans General Hospital Department of Biostatistics Task Force for providing the consultation of data analysis.

## Author contributions

**Conceptualization:** Hung-Ming Wu, Chih-Ping Chung, Yung-Yang Lin.

**Data curation:** Hung-Ming Wu, Yung-Yang Lin.

**Formal analysis:** Hung-Ming Wu, Yung-Yang Lin.

**Investigation:** Hung-Ming Wu, Yung-Yang Lin.

**Methodology:** Hung-Ming Wu, Yung-Yang Lin.

**Writing – original draft:** Hung-Ming Wu.

**Writing – review & editing:** Chih-Ping Chung, Yung-Yang Lin.
